# The Role of Parvalbumin Interneurons in Neurotransmitter Balance and Neurological Disease

**DOI:** 10.3389/fpsyt.2021.679960

**Published:** 2021-06-18

**Authors:** Lailun Nahar, Blake M. Delacroix, Hyung W. Nam

**Affiliations:** Department of Pharmacology, Toxicology, and Neuroscience, Louisiana State University Health Sciences Center, Shreveport, LA, United States

**Keywords:** parvalbumin expressing fast-spiking interneurons, excitatory/inhibitory balances, GABA-glutamate interaction, GABAergic disinhibition, neurological disease

## Abstract

While great progress has been made in the understanding of neurological illnesses, the pathologies, and etiologies that give rise to these diseases still remain an enigma, thus, also making treatments for them more challenging. For effective and individualized treatment, it is beneficial to identify the underlying mechanisms that govern the associated cognitive and behavioral processes that go awry in neurological disorders. Parvalbumin fast-spiking interneurons (Pv-FSI) are GABAergic cells that are only a small fraction of the brain's neuronal network, but manifest unique cellular and molecular properties that drastically influence the downstream effects on signaling and ultimately change cognitive behaviors. Proper brain functioning relies heavily on neuronal communication which Pv-FSI regulates, excitatory-inhibitory balances and GABAergic disinhibition between circuitries. This review highlights the depth of Pv-FSI involvement in the cortex, hippocampus, and striatum, as it pertains to expression, neurotransmission, role in neurological disorders, and dysfunction, as well as cognitive behavior and reward-seeking. Recent research has indicated that Pv-FSI play pivotal roles in the molecular pathophysiology and cognitive-behavioral deficits that are core features of many psychiatric disorders, such as schizophrenia, autism spectrum disorders, Alzheimer's disease, and drug addiction. This suggests that Pv-FSI could be viable targets for treatment of these disorders and thus calls for further examination of the undeniable impact Pv-FSI have on the brain and cognitive behavior.

## Introduction

GABAergic interneurons play an essential role in maintaining a fine-tuned excitation-inhibition balance in the brain. GABAergic interneurons are diverse with more than 20 types of interneurons that can be classified based on anatomical, molecular, and physiological properties (1–3). The anatomical classification of interneurons is based on targeting pyramidal cells or other interneurons. Pyramidal cells targeting interneurons are further divided into their target location to synapse including chandelier cells, basket cells, and dendrites. The molecular classifications of interneurons are divided into five subgroups based on the expression of specific molecular markers: (1) parvalbumin (Pv) in chandelier and basket cells; (2) somatostatin (SOM); (3) neuropeptide Y (NPY); (4) vasoactive intestinal peptide (VIP); and (5) cholecystokinin (CCK). Although, these interneurons can be similar in their morphology and connectivity properties, their intrinsic electrophysiological and biochemical properties is a major disparity that also sets them apart from each other (4). Moreover, the physiological classification of interneurons is identified as six main types of interneurons (1). Fast-spiking interneurons (FSI) fire at a continuous high frequency >50 Hz at steady-state, which is considerably higher than the firing rates of principal cells. After a large hyperpolarization burst, FSI prolong spikes during the continuous, delayed, and stuttering stage. Non-fast spiking interneurons (NFSI) display no apparent increase of frequency in the inter spike interval at steady-state. Adapting interneurons (ADI) display a significantly increased inter spike intervals at burst stage and maintain steady-state during the continuous and delayed stage. Irregular spiking interneurons (ISI) display an irregular inter spike interval at burst and continuous stage. Intrinsic bursting interneurons (IBI) produce a stereotypical burst of two or more spikes on a depolarization followed by a hyperpolarization potential during the continuous stage. Accelerating interneurons (ACI) display a decrease in the inter spike interval at steady-state and delayed stage. Although, these classifications provide only partial knowledge of each interneuron, a more comprehensive understanding involving multiple anatomical and functional criteria imposes a practical application for studying the etiologies and pathologies of neurological and psychiatric disease.

Moreover, the corticostriatal circuitry, which consists of the medial pre-frontal cortex (mPFC) and the striatum, plays an essential role in cognition, learning, and reward-seeking behaviors. Proper cognitive functioning and reward behavior rely on the corticostriatal circuit, and therefore, cortical neurons, and medium spiny neurons (MSNs). However, the molecular mechanisms that govern these processes still require investigation. Researchers have established that the mPFC is important for executive and cognitive functioning, including cue-mediated behaviors. The striatum, on the other hand, contains neuronal hubs that are crucial for integrating cognitive and contextual information, and for processing motivation and reward. Indeed, interneurons allow for communication within circuits but possibly also between circuits that subsequently give rise to cognitive behaviors. Therefore, this review discusses the role of parvalbumin-containing fast-spiking interneurons (Pv-FSI) in the cortex, striatum, and hippocampal networks and how Pv-FSI functions are impaired in brain diseases.

Pv-FSI make up ~ 50% of the cortical interneuron population of the basket and chandelier cells that innervate pyramidal cells in the cortex. Intricacies and complexities of Pv-FSI highlight the need of further delineation of their function considering the growing evidence of the diversity within these interneurons, especially in terms of their morphological and synaptic properties. An ability of these GABAergic Pv-FSI that appears to be universal across cortical regions is controlling spike timing in neighboring pyramidal cells, which are excitatory glutamatergic neurons (5). While these interneurons may have inhibitory postsynaptic effects on excitatory neurons, Pv basket cells strongly connect to other Pv cells to disinhibit their functioning such that there is decreased synaptic inhibition of excitatory neurons (6).

As many psychiatric and neurological diseases endure glutamate imbalance or excitotoxicity, Pv-FSI serve as a promising target because these interneurons are powerful regulators of pyramidal neuron activity. Nonetheless, they appear to be the most vulnerable interneurons across psychiatric disorders that involve cognitive failure, including schizophrenia, autism spectrum disorders, Alzheimer's, and drug addiction (7). However, the contribution of interneurons in circuits involved in psychiatric conditions have not been fully established. Thus, identifying how Pv-FSI is key players in health and disease and defining their exact function in specific brain regions and networks is crucial, especially because although, a variety of cognitive-behavioral deficits are exhibited across different mental illnesses, there are some overlaps between them as well.

## Pv-FSI Expression in the Brain

### Pv-FSI Expression in the Cortex

The cortex has numerous dynamic functions in cognition, and behavior controlled by the regulation of neuronal signals to many other brain regions. Specific functions of cortical neurons are typically determined by their location in the cortex. One region of interest is the prefrontal cortex, located in the anterior frontal lobe in humans, which allows for elaboration of thoughts and converting thought into motor and non-motor activity (8). The cortex contains many types of GABAergic neurons that inhibit cortical pyramidal cells (9). Fast-spiking interneurons are one type of GABAergic interneuron that is particularly of note because of the large effect they have on cortical function. Although, Pv-FSI are comprised of <2% of total neuronal population in the mPFC, Pv-FSI make up the largest class of GABAergic interneurons in the neocortex accounting for about 40% of such neurons (10). Pv interneurons are in all layers except for L1, indicating specific cognitive and behavioral functional relevance of these interneurons in the cortex. Strikingly, even the most abundant morphologically distinct subtype of Pv interneurons, called Pv basket cells, can also exhibit functional differences despite being from the same cortical layer (6).

Of the two main types of Pv-FSI projections in the cortex, Pv basket cells innervate soma, and proximal dendrites whereas, Pv chandelier cells innervate axons of postsynaptic pyramidal cells (11). These interneurons are known for their low input resistance, high-amplitude rapid after-hyperpolarization, and fast-spiking phenotype, all of which make them unique to other neurons in that they can fire many action potentials quickly, and why they were termed “fast-spiking.” Since Pv-FSI synapse onto almost all nearby pyramidal cells, Pv-FSI plays a critical role in cortical inhibition (11). It has been shown that through this fast-spiking behavior, generally all Pv-FSI can control the timing of spikes in excitatory neurons and modulate the glutamatergic activity of pyramidal neurons in the mPFC (12–15).

Cortical layer 2/3 (L2/3) is unique in that pyramidal neurons have sparse firing rates and provide input to Pv-FSI at far higher rates than to neighboring pyramidal neurons. Using *in vivo* two-photon targeted whole-cell recordings in genetically labeled neurons in L2/3 of mice, researchers identified that the action potential of a single presynaptic pyramidal neuron evokes unitary excitatory postsynaptic potentials (EPSPs) that temporally and precisely evoke action potentials in Pv-FSI compared to the little effect of unitary EPSP firing onto other neighboring excitatory neurons and interneurons (16). Remarkably, Pv-FSI showed to have a higher excitatory synaptic gain in which the number of additional action potential in Pv-FSI were higher than other neurons. This work demonstrates a profound effect of a single pyramidal neuron action potential on GABAergic Pv-FSI that consequentially go on to inhibit the local excitatory network in a precise and timely manner, suggesting that a single spike evoked disynaptic inhibition is a mechanism of modulating and maintaining cortical signaling. Although, done *in vivo*, more studies must examine the behavioral and circuit effects of Pv-FSI evoked by unitary EPSPs by a single excitatory neuron.

In addition, the role of Pv-FSI in the mesocortical pathway is not fully understood, however, the expression of D1 and D2 receptors on their cell surface suggests that Pv-FSI are affected by dopamine (17, 18). Some studies have shown a strong influence of dopamine upon cortical (19) and mesocortical Pv-FSI through dopamine signaling, which can in fact trigger inhibition of principal cells in the cortex (20). More studies will need to be done to elaborate the dopamine pathways involving Pv-FSI in the cortex.

### Pv-FSI Expression in the Hippocampus

The hippocampus is critical for the consolidation of both short-term and long-term memories. Pv-FSI play a major role in the hippocampus although, remarkably, Pv-FSI make up a small percentage of the neuronal population there. In the CA1 region of the hippocampus, Pv-FSI account for 24% of the GABAergic neurons and about 2.6% of the total neurons in that area (21, 22). Pv-FSI are critical in memory formation and information processing. After novel experiences, Pv-FSI coordinate and stabilize CA1 neuron communication (22). They are also involved in massive lateral inhibition within the dentate gyrus (23). These interneurons tend to innervate principal cells in the deeper layers of the hippocampus rather than those of superficial layers of the stratum pyramidale (21). Pv-FSI give rise to gamma oscillations which are essential to normal brain functioning and cognition, specifically, cortical information processing (24, 25). Periodic optogenetics stimulation of Pv-FSI in the hippocampus is sufficient to generate gamma oscillations in a cortical network (26) and a deficiency in Pv also show an increase in gamma oscillations (27). It is still questionable, however, whether or not Pv-FSI are necessary to elicit gamma oscillations and what effect these oscillations have on information processing (28). More research needs to be done to elaborate the unique role of Pv-FSI in the production of gamma oscillations, and the purpose of gamma oscillations themselves.

### Pv-FSI Expression in the Striatum

The striatum is important for reward-seeking, motivation, and motor function. In the striatum, Pv-FSI play a role in reward-seeking behavior and sensorimotor signaling. Pv-FSI synapse with medium spiny neurons (MSNs) (29) but the effect of Pv-FSI functioning in the striatum is uncertain, considering there are many striatal interneurons. Pv-FSI are found in the nucleus accumbens (NAc) as well, where they make up about 2–3% of the neuronal population. There, they provide feedforward inhibitory control of MSNs, which are believed to be involved in drug abuse. However, it has been shown that Pv-FSI are not involved in amphetamine-stimulated dopamine release in the NAc (30). In addition, Pv-FSI target about 25–75% of MSNs, especially striatonigral MSNs and some striatopallidal MSNs. Reduced GABAergic signaling by Pv-FSI in the striatum is sufficient to reduce dystonic movements in mice. The striatal Pv-FSI express AMPA glutamate receptor GluA2 subunit, whereas MSNs do not, making Pv-FSIs a great target specific to the striatum (31). Since each Pv-FSI influences hundreds of MSNs around them, it is believed that they have a broader function in the striatum rather than a localized response in specific brain regions (32).

## Pv-FSI Regulation in Neurotransmission

### Pv-FSI Mediates GABA-Glutamate Balance

In the cortex, the activity of excitatory pyramidal neurons (glutamatergic) are molated by inhibitory interneurons (GABAergic). Inhibitory interneurons control the firing of postsynaptic neurons by preventing feedforward excitation from other afferent neurons (11). Their regulation of glutamatergic neurons *via* forward feedback inhibition leads to a broader array of firing patterns instead of only having the typical neurons' all-or-none characteristic (11). Excitatory-inhibitory (E/I) balance must be stable in order for feed-forward inhibition and control by Pv-FSI to occur efficiently. As such, there is evidence that Pv-FSI are important in E/I balance in the hippocampus as well. Hippocampal Pv-FSI play a key role in the development of temporal lobe epilepsy. The E/I balance in the dentate gyrus is altered in this disease. In epileptic patients, there is a major loss in the number of PV-FSI in this area, reducing inhibition of granular cells. This then induces hyperexcitation of neurons in the hippocampus, particularly in sclerotic regions, resulting in epileptic seizure activity (33).

Many research up to date have substantiated that Pv-FSI play a significant role in the pathogenesis of schizophrenia by disrupting glutamate signaling and hence GABA-glutamate balance. Pv-FSI are recruited by glutamatergic pyramidal cells and their proper function is dependent upon this process. Defects in this process could be the one of the causes of psychiatric disease like schizophrenia (7). The glutamate hypothesis of schizophrenia states that glutamate neurotransmission, as opposed to or even alongside dopamine neurotransmission, plays a central role in the development of schizophrenia (34). Pv-FSI are sensitive to NMDA glutamate receptor antagonists and this receptor inhibition has been shown to cause a schizophrenia-like syndrome in humans. Mutating these NMDA glutamate receptors yield a similar disease pattern in mice. There is a direct connection between a reduction in glutamatergic excitation of Pv-FSI and glutamate decarboxylase 67 (GAD67) levels in these interneurons, decreasing their GABAergic ability (7). In addition, there is evidence that deficits in glutamatergic activation of Pv-FSI may also play a role in the development of autism spectrum disorders. The current understanding of Pv-FSIs' role in these disorders is limited. Therefore, future research focusing on E/I balance involving Pv-FSI in diseases like schizophrenia, autism spectrum disorders, and epilepsy could further our understanding of these diseases, and potentially aid in treating them.

### Pv-FSI Mediates Disinhibiton Between GABAergic Neurons

Pv-FSI activation projecting onto the NAc affects GABAergic MSNs (35–37). Since a single Pv-FSI inhibits an estimated number of 100 MSNs (38), Pv-FSI can certainly have a great impact on the communication of this circuitry. In fact, activation of Pv-FSI in the NAc result in behavioral changes to reward (37) and cocaine addiction (39). Electrophysiology experiments have proved the vast GABAergic and fast-spiking activity of Pv-FSI. In addition, *in vivo* neural recordings and optogenetics demonstrate that if Pv-FSI are suppressed, then consequently, these interneurons would reduce MSN firing (35). One study found that the most powerful inhibitory input of Pv-FSI is autaptic transmission, stimulating the same neuron onto itself, in the neocortical layer V (40). Use of optogenetics showed the strength of autaptic transmission modulates γ-oscillations and prevents high-frequency bursts of spikes. Researchers found that autaptic transmission of Pv-FSI is ~3-fold stronger than the synaptic inhibition onto pyramidal neurons and ~2-fold larger than interactions between Pv-FSI neurons. Moreover, this study found that Pv cells with strong autaptic transmission produce a weaker synaptic output onto other Pv cells and vice versa, thus defining a cortical network of connectivity control mediated by Pv-FSI. Overall, this finding supports that fast autaptic self-inhibition of Pv-FSI contributes to the degree of synchronization with γ-oscillations.

### Pv-FSI Mediates Dopamine Release

Reduced numbers of Pv-FSI in the ventral hippocampus can cause hippocampal hyperactivity and have a significant downstream effect on dopamine signaling (41). While the mechanism of this association has not been elucidated yet, this hyperactivity has been shown in both human schizophrenia patients and mouse models (41). Amphetamine drug-induced dopamine release has been shown to increase the activity of Pv-FSI (42). Pv-FSI activity has also been shown to increase in periods of cocaine withdrawal. Ethanol consumption however, decreases Pv-FSI activity, and facilitates activation of D1-like and D2-like receptors on the cell surfaces of these interneurons (42), and increases dopamine release in the NAc. Interestingly however, some also report no significant changes in dopamine release directly related to Pv-FSI activation (43). Other studies also have shown a strong influence of dopamine upon cortical Pv-FSIs. Mesocortical stimulation of cortical Pv-FSI through dopamine signaling can in fact trigger inhibition of principal cells in the cortex (19, 20). Since dopamine excess is a known factor in the pathogenesis of schizophrenia, it's possible that Pv-FSI play a role in that of other psychiatric disorders that have dopamine circuitry as an underlying mechanism, such as in substance use disorders. More research should be done in this area to delineate the dopamine pathways involving Pv-FSI neurotransmission to gain a better understanding of pathogenesis of these diseases.

## Pv-FSI Dysregulation and Diseases

### Pv-FSI Regulation in Human Neurological Disease

Dysregulated Pv-FSI indicates E/I disturbances in psychiatric disorders and their complexities because Pv-FSI can affect multiple inter-neuronal cell types and do not appear to be a consequence of disease treatment or progression. Rather, disturbances in Pv-FSI are reported to be a hallmark of disease progression, for example, of schizophrenia, whereas, glutamate and dopamine are implicated in the pathophysiology and treatment of schizophrenia (44, 45). Pv-FSI provide inhibitory control of cortical and subcortical circuits, disruptions of which are known to lead to glutamatergic and dopaminergic dysfunction and in turn leads to the symptoms of schizophrenia. A deficit in Pv-FSI functionality is possibly due to decreased inhibitory Pv-FSI control over pyramidal cell activities and or a reduction in the brain connectivity of large brain networks.

Moreover, a failure in coordinated information processing between brain regions has been suggested to account for a wide range of deficits in schizophrenia. Patients with schizophrenia show a decreased expression of GAD67 in the dorsolateral PFC (dlPFC), which may contribute to the cognitive symptoms of schizophrenia. In addition, schizophrenia patients relative to comparison subjects have almost 50% lower GAD67 protein levels in Pv axon terminals, further, suggesting that less GABA is synthesized and released by these cells (46). Abnormal gamma oscillations are also observed in schizophrenia patients as early as in their first psychotic episode (47), and are potentially due to the decreased levels of cortical GAD67 and consequent decrease GABA synthesis in those (48). All this may also contribute to the cause of many cognitive working-memory deficits and possibly other symptoms in those patients as well.

Other modes of disrupted cell communication like impaired Pv-FSI signaling upstream of Pv-FSI activity can indirectly contribute to etiologies of disorders. For instance, mechanisms governing cortical disinhibition can also go awry when the normal synaptic inhibition of pyramidal neurons by Pv-FSI is reduced due to these Pv-FSI being suppressed or inhibited by other interneurons. For example, the density of excitatory synapses were found to be lower and selectively on Pv interneurons in the dlPFC of schizophrenia patients. These results also corresponded to deficits in GAD67, suggesting a more precise and cell-type-specific mechanism of cortical dysfunction and cognitive deficit that occurs in schizophrenia (49).

Evidence of GABA dysfunction occurring in schizophrenia patients can be dated back to earlier neuropathological studies that have reported reduced GABAergic interneurons in the hippocampus of human schizophrenia patients and moreover, a substantial difference in the relative density of Pv-positive neurons in the hippocampus compared to normal controls (50). In addition, a decrease in Pv expression and Pv-FSI in the CA1 region of the hippocampus is exhibited in Alzheimer's patients (51). This decrease in Pv expression could lead to the pathogenesis of Alzheimer's *via* an increase in glutamatergic excitotoxicity in the hippocampus (51). Pv-FSI have also been implicated in the development of autism spectrum disorders. Specifically, a deficiency of Pv-FSI in the striatum of mouse models reflected the importance of these interneurons in autism spectrum disorders (52, 53). The downregulation of these neurons is hypothesized to be caused by a mutation in pre-stress conditions postnatally or environmental stressors prenatally. It is believed that the downregulation of Pv in these interneurons helps to monitor and control the E/I balance, but at the expense of developing a phenotype of autism (52). How Pv-FSI may monitor and maintain the brain in a healthy state as opposed to falling into a vulnerable or diseased state is yet to be determined. Possible mechanisms of Pv-FSI dysfunction and Pv-implicated diseases focus on the malfunction of certain genes that are responsible for cell functions that are characteristic of Pv functioning. For instance, as an important and proper functioning of Pv cells is to repolarize fast and relies on calcium sequestration, it is possible that genes responsible for calcium signaling are defective, causing Pv-FSI to malfunction altogether.

Another interesting aspect of Pv interneurons in the brain is the connections they form with cellular structures. Several studies have shown that Pv-related psychiatric diseases induce changes in cell structures like the extracellular matrix (ECM) and perineuronal nets (PNN) across several brain regions (54). PNNs preferentially surround Pv-FSI both are subject to change after exposure to stress, therefore possibly impairing the Pv-networks throughout the brain. A review of studies that have examined these relationships using many different behavioral models of stress and at different time points (prenatal, early and late adolescence, adulthood etc.,), levels of exposure, and trajectories, have consistently shown that stress results in changes in PNN-Pv density (54).

### Pv-FSI Expression and Sexual Dimorphism

Differences in Pv expression between sexes is a unique facet of Pv-FSI and this implores for more investigation to understand Pv-FSI-related brain diseases that are sexually dimorphic. There is a greater deficit in the relative density of Pv-positive neurons in the hippocampus of male schizophrenic patients compared to that of female patients (50). Moreover, sexual dimorphism was observed in the developmental effects of Pv-FSI in the mPFC (55) and striatum (56). As disturbances in depression and reward-seeking behaviors such as substance abuse and addiction also present gender as a demographic difference, it is imperative to research how Pv-FSI attributes to these differences. This can provide further insight into how Pv-FSI can contribute to the cognitive and behavioral dysfunction and the neurochemical imbalance that occurs in in these diseases and therefore also provide avenues for individualized treatment methods based on gender.

Further, study is required to clarify the function of Pv-FSI not only in specific brain regions but also its function in an age- and sex-dependent manner. One study found that early life stress decreases the number of Pv-FSI in female mice, indicating these interneurons are sensitive to stress during development, but mainly for females (55). Females are about twice as likely as males to have a mental illness related to stress, lending a possible explanation for the decreased number of Pv-FSI in brains of such females (57, 58). Moreover, female unpredictable chronic mild stress (UCMS) mice were affected more severely than male mice and this behavioral finding was in conjunction with greater increases in Pv mRNA expression, Pv neurons, and increased NMDAR activation within those neurons in female PFC. This study found significant effects of gender on the glutamatergic neurotransmission onto prefrontal Pv-FSI of mice undergoing UCMS (59). Further, studies have elucidated a possible mechanism for this, showing that chronic chemogenetic activation of prefrontal Pv sufficiently induces anxiety-like phenotypes in female mice whereas acute activation does not (60). One study found that administration of 17-beta-estradiol to rats without ovaries increased levels of Pv expression and GAD67 expression, but high estrogen levels can cause dysfunction in the hippocampus (61, 62). Testosterone both induces anti-inflammatory cytokine release and represses pro-inflammatory cytokine release, and thereby serves as a protective effect against possible damages to the Pv-FSI in the brain (50).

## Pv-FSI Regulation Changes Behaviors

### Pv-FSI Regulation in the mPFC Modulates Cognition

Many researchers have conducted studies that suppress Pv behavior in certain brain regions, and even in certain neurons. As a lot of cognitive functioning relies on information processing, it is important to assess the circuits involved and how Pv-FSI plays a role. Pv-FSI themselves are specialized cells but, there are specific proteins in Pv-FSI that are essential to specific behavioral functions (Table 1).

**Table 1:** The role of Pv-FSI in neurological and psychiatric disorders.

	**Cortex**	**Hippocampus**	**Striatum**
Schizophrenia	• NR1 deletions in interneurons cause schizophrenia-like symptoms due to lower numbers of NMDA receptors (34). • E/I regulation *via* Pv-FSI optimize info processing; disruption is sufficient to cause psychiatric disorders (11). • Early life stress reduces numbers of Pv+ interneurons in cortex, more so in females than males (55). • Dopamine signaling activates Pv-FSI. Tested *via* whole cell patch-clamp study (19). • Pv Cre; ErbB4^−/−^ mice show deficits in working memory (63). • Latent inhibition paradigm, electrophysiology, immunohistochemistry (64). • PCP postnatally in rats selectively reduces Pv interneurons (65). • Pv+ interneurons are also D4+ *via* immunohistochemistry (17). • Modulation of Pv-FSI in the neocortex *via* optogenetics affects gamma oscillations (28). • Pv-Cre; ErbB4^−/−^ mice, Electrophysiology recordings; immunostaining (66). • Chemogenetics, drug applications (ketamine) and CNO; Whole-cell patch-clamp recordings, Calcium imaging of Pv-FSI; Restraint stress behavior experiments (67). • Assessment of Pv and synaptic density in the DLPFC in human schizophrenia subjects. Fluorescent immunohistochemistry and confocal microscopy showed significantly lower Pv levels in subset of Pv interneurons and fewer excitatory synaptic inputs onto Pv cells (49).	• Deficit in the relative density of Pv-positive neurons. Tissue from post-mortem schizophrenic patients show men have greater reductions than female schizophrenic patients (50). • Use of NMDA pharmacological targets: Ketamine and PCP reduces Pv density (68–70). • shRNA-silenced parvalbumin genes induce hyperactivity in ventral hippocampus (41). • Tetanus-toxin-induced lesions of Pv+ interneurons and role in social memory (21). • Latent inhibition paradigm, electrophysiology and, immunohistochemistry (64). • NMDAR in Pv FSI (NR1PVCre–/–); gamma and theta oscillations; impaired spatial working, and spatial recognition memory (71). • E2 signaling in TMT-treated rats upregulates Gad67 and parvalbumin production in Pvv+ interneurons. Sexual dimorphism (61).	
Substance use disorders	• Ethanol decreases Pv+ transmission shown *via* microdialysis and operant conditioning of mice; plays role in reward-seeking behavior (43). • E/I regulation *via* Pv-FSI optimize info processing; disruption is sufficient to cause psychiatric disorders (11).		• Regulation of interneurons *via* dopamine signaling in addiction (42). • Pv-Cre transgenic mice have reduced voluntary ethanol consumption (72). • NAc-specific Pv-FSI activation influences cocaine and methamphetamine reward properties. Conditioned place preference (CPP) behavioral paradigm (73). • Express tetanus toxin light chain (TeLC) selectively in Pv+ interneurons to silence Pv-FSI. Disrupts locomotor sensitization and conditioned place preference in amphetamine intoxication (30). • Locomotor sensitization and Conditioned Place Preference (37).
Autism spectrum disorders	• Using CRISPR/Cas9 and iPSC to tag Pv-FSI in development (53). • Modulation of Pv-FSI in the neocortex *via* optogenetics affects gamma oscillations (28). • Chemogenetics, drug applications (ketamine), and CNO; Whole-cell patch-clamp recordings, Calcium imaging of Pv-FSI; Restraint stress behavior experiments (67).	• Tetanus-toxin-induced lesions of Pv+ interneurons and role in social memory (21). • E2 signaling in TMT-treated rats upregulates Gad67 and parvalbumin production in Pv+ interneurons. Sexual dimorphism (61).	• Cntnap–/– mice showed no loss in number of Pv+ interneurons, but a decrease in expression of parvalbumin in Pv+ interneurons (52).
Alzheimer's disease		• Comparison of 3xTg-AD (Alzheimer's disease) mice with non-Tg mice showed decrease Pv levels in Pv+ interneurons in CA1 region of hippocampus in AD mice (51). • Tetanus-toxin-induced lesions of Pv+ interneurons and role in social memory (21). • E2 signaling in TMT-treated rats upregulates Gad67 and parvalbumin production in Pv+ interneurons. Sexual dimorphism (61).	
Depression/stress	• Sexual dimorphism: increased extracellular signal-regulated kinase 1/2 (ERK1/2) activity, pERK expression in Pv neurons of females undergoing unpredictable chronic mild stress (UCMS) (59). • Acute and chronic activation of Pv+ cells by chemogenetics (DREADD; CNO) reduces prefrontal activity. Only the chronic activity results in increased anxiety-like behaviors in females (60).	• Stress-induced changes in ECM/PNN and Pv+ interneurons (54).	

Although, cortical neurons are mainly excitatory glutamatergic cells with many projections, GABAergic Pv-FSI in the mPFC also target and influence glutamate signaling. Pv-FSI and schizophrenia research focusing on glutamate signaling found that ablation of NMDA glutamate receptors in Pv-FSI impairs spatial working memory in mice (71). In addition, NMDA glutamate receptor blockade by phencyclidine (PCP) has been suggested as a model to examine the pathophysiology of schizophrenia (65, 68). Moreover, administering PCP postnatally in rats selectively reduces Pv-FSI in the cortex in adulthood (65).

Pv-FSI is also involved in regulating depression-like behavioral output (74) by targeting glutamatergic signaling. Ketamine administration results in the rapid release of glutamate primarily in the PFC. Efforts to understand this mechanism and the behavioral antidepressant effects calls for the consideration of interneurons. Indeed, knocking down the GluN2B (NMDA glutamate receptor subtype 2B) in the mPFC of Cre-Pval mice occludes ketamine's antidepressant behavioral effects (74). Contrary to these results, however, is evidence that ketamine enhances Pv-FSI activity and that these interneurons are protective against stress-induced synaptic impairments. Inhibiting them eliminates this effect (67). Future studies can benefit from a more in-depth analysis of neuromodulators and proteins downstream of receptor activity to elucidate the exact electrochemical signals of Pv-FSI to cognition and behavior. An interesting question is whether Pv-FSI preferentially mediates glutamate signaling through NMDA glutamate receptor vs. metabotropic glutamate receptors because NMDA glutamate receptor are more fast-acting ionic channels that can synchronize with fast-spiking activity of Pv-FSI.

Myelination of Pv-FSI plays an important role for the establishment and maintenance of Pv-FSI-mediated GABAergic inhibition in cortical sensory processing (75). Defects in myelination of Pv-FSI diminish their high firing frequency as well as delay their action potentials. This ultimately decreases their connectivity with excitatory neurons, contributing to a strong imbalance of excitation and inhibition. These deleterious effects of myelination defects and excitation-inhibition imbalance are associated with impairments in whisker-dependent texture discrimination in mice, suggesting that myelination of Pv-FSI may be critical for the outcome of sensory neuronal processing. Whether or not this is a relevant phenomenon of sensory processing in humans is not known, but may be worth investigating since many psychiatric diseases such as autism and sensory processing disorder display impairments in sensory processing.

In addition, reduction of GABAergic transmission from Pv-FSI primarily impacts to the behavioral phenotypes observed in schizophrenia (3). Pv-FSI inhibition of pyramidal neurons are reduced due to lower GAD67 mRNA expression and lower GAD67 protein, indicating less GABA synthesis (46, 76). Researchers have specifically targeted the glutamate decarboxylase 1 (GAD1) gene-encoded 67-kDa protein isoform of GAD67 as a hallmark of schizophrenia in Pv-FS sin transgenic mice. Mice with GAD1 knocked down in Pv-FSI exhibited decreased GABAergic synaptic transmission and behavioral disruptions that align with phenotypes of schizophrenia, such as in sensorimotor gating deficits, fear, and novelty seeking (3). MAM (Methylazoxymethanol acetate) G17 is another verified animal model of schizophrenia which administers mitotoxin, MAM, on gestational day 17. This animal model of schizophrenia shows a region-specific reduced expression of Pv-FSI in the mPFC and ventral subiculum of the hippocampus (64). In addition, under a latent inhibition paradigm, rats have a reduction in gamma bands in these brain regions. This observation implicates a correlation between the deficit in neuronal activity and behavioral impairment that can explain the reduced GABAergic signaling and hypo functioning of the related brain regions that occur in schizophrenia (64). Utilizing tools to look at single-cell sequencing and mRNA levels across different brain regions would help to elucidate which cells are more vulnerable to different diseases of Pv-FSI impairment.

Abnormal protein composition in Pv-FSI have drastic behavioral effects. For instance, deletion of Erbb4 in Pv-FSI results in impaired prepulse inhibition (PPI) and working memory (66, 77). Such a change in molecular composition may contribute to behavioral effects because the Erbb4 protein affects the inherent molecular function of Pv-FSI. Reduced Erbb4 in Pv-FSI causes cortical GABAergic neurotransmission to be impaired as well as Pv microcircuit communication due to fewer synapses onto pyramidal cells and less innervation from pyramidal cells (77). These studies highlight the importance of the genetic component that drives cognitive and behavioral function and is an open area of research in the field.

### Pv-FSI Regulation in the Hippocampus Modulates Cognition

Studies on cognition has stemmed from disease models in which there are cognitive dysfunctions. As such, non-competitive antagonists of the NMDA glutamate receptor such as the PCP and ketamine have been used in psychiatric research. Modulating glutamate activity in this way demonstrated changes in Pv-FSI in the hippocampus. Studies using animal models to induce cognitive deficits of schizophrenia by administration of PCP showed that sub-chronic PCP in rats also reduces the density of Pv-positive neurons in the hippocampus with profound deficits in the dentate gyrus and CA2/3 regions (68). Ketamine administration to rodents have also demonstrated diminished Pv density similar to those found in schizophrenia patients (69, 70). Moreover, to understand cognitive dysfunction in schizophrenia, sub-chronic administration of PCP to rats showed it induces deficits in operant reversal learning and has a correlation with reduced Pv expression in the hippocampus (68). Therefore, Pv in the hippocampus seems to play a role in the cognitive dysfunction in schizophrenia, and the pathophysiological mechanism underlying it may be due to the localized glutamatergic signaling. Future studies need to elucidate the importance of GABAergic Pv neurons and glutamate signaling and how this contributes to cognition directly in healthy and diseased models.

Moreover, it is not just the function of Pv-FSI in the hippocampus that plays a role in cognitive function, but also the molecular effects of Pv-FSI as well; thus, some studies looked at the impact of reduced Pv-FSI in the hippocampal neuronal networks (78). For instance, in the hippocampus, an absence of Pv facilitates repetitive GABA release that can increase the inhibitory effects of Pv-FSI and thus affect the higher cognitive functions associated with gamma oscillations (27). Pv-FSI, being the important and necessary contributors for the generation of gamma oscillations, are a great target for understanding information processing in hippocampal networks. Another example is the generation of conditional Cre-Pval-*GluR-A*^*f*/*f*^ mice which results in Cre-mediated ablation of the GluR-A gene in Pv-positive cells. These mice exhibited abnormalities in spatial working memory, novel object exploration, and the response to changes in the spatial relationships among multiple objects (78). Pv-FSI studies may suggest a role in anxiety as well. When mice were employed the elevated plus maze paradigm, investigators found that specific activation of Pv-FSI and enhancing their function in the dentate gyrus (DG; important for learning and memory) produces an anxiolytic effect but no effects on depression (79). These data further support the importance of the unique physiological properties of Pv for proper molecular functioning of cells that allow them to form networks and give rise to cognition or behavior.

The hippocampus is important for understanding environmental cues and plays a role in encoding these into memory. Social memory involving the hippocampus is one of the cognitive processes that is impaired in psychiatric disorders like Alzheimer's, schizophrenia, and autism with potential links to altered Pv-FSI physiology (21, 80–82). The ventral hippocampus has been found to regulate social memory and Pv-FSI play an essential role in discriminating novel experiences from familiar ones (79, 82). Specifically, the Pv-FSI in the CA1 region, where a quarter of GABAergic cells are Pv-FSI engage in the retrieval stage of social memory as opposed to memory encoding, as observed by the hyperexcitation of these interneurons that impaired the ability of mice to recognize familiar mice (21, 83). In addition, Pv-FSI modulation of gamma oscillations play a role in social discrimination (21). A lack of CA1 Pv-FSI firing coincided with a blockade of contextual fear memory consolidation and a loss of stabilization of CA1 network communication patterns for optimal learning. Delta and theta oscillations were also noted after contextual fear conditioning (84). Pv-FSI are sufficient to stabilize CA1 communication networks for hours (22). Therefore, studying Pv-FSI in the largely populated CA1 region of the hippocampus seems to be a promising direction for identifying the pathophysiology and related mechanisms for disease progression of neurological diseases. Further, studies can modulate the hyperexcitation of these interneurons to evaluate therapeutic potential.

### Pv-FSI Regulation in the Striatum Modulates Reward-Seeking

Although, the striatum alone has been deeply studied and established as a part of the reward system, manipulating the striatal circuits and neuronal networks it is connected to has been a new area of focus to resolve questions in this field. The corticostriatal circuitry plays an essential role in coordinating and regulating neuronal excitability during reward-seeking behaviors. Moreover, the mPFC is important for the expression and extinction of cue-mediated behavior and is regulated by cortical neurons, while the NAc is an essential hub that integrates cognitive, contextual, and affective information and is mediated by MSNs (85, 86). Glutamate dysregulation in this circuit plays a role in the reward-seeking behaviors as well as the compulsive behaviors that characterize addictive behaviors as well as its development.

Although, glutamate dysregulation is a possible explanation for cognitive dysfunction and consequent compulsive reward-seeking during substance abuse, there is more to the story of this molecular mechanism since the contribution of interneurons in glutamate circuitries have not been fully established. Pv-FSI, specifically, have been implicated in studies on cognition and reward-seeking (5, 43). Even though Pv-FSI constitute as little as only 2% of the neuronal population in the mPFC and NAc, they are adept in controlling the glutamatergic outputs of pyramidal neurons in the mPFC (13), while also modulating GABAergic MSNs in the striatum (35, 37). Moreover, a single Pv interneuron inhibits ~100 MSNs (38); Pv-FSI exert profound effects downstream onto effector cells and thus, can be a major source of changes in its microcircuit causing glutamate dysregulation and subsequently cognitive dysfunction and behavioral dysregulation. This provides an exciting direction for future studies as an alternative to the current inefficient treatment and research strategies for substance abuse.

Recent developments in the field of reward and drug addiction have been to elucidate the role of interneurons and their involvement in brain circuitry associated with impaired cognition and behavior in drug abuse. Moreover, studies modulated Pv-FSI activity to see how it alters reward-seeking behaviors for natural rewards like sucrose and drugs of abuse like ethanol (43, 72) amphetamine (37), and cocaine (39, 73). Operant conditioning paradigms have served as an important tool to assess and understand reward behavior. It has been reported that mPFC-specific Pv-FSI activation upon demonstration of cues associated with reward significantly accelerated the extinction of reward-seeking behavior (13), while NAc-specific Pv-FSI activation also influences cocaine (39) and methamphetamine reward properties (37, 73). Upon the administration of acute amphetamine, Pv-FSI display robust firing in the striatum (87). Pv-FSI in the NAc are necessary for the behavioral responses that occur following chronic amphetamine use and may also influence the behavioral adaptations associated with psychostimulant drugs of abuse. Alcohol inhibits GABA release from interneurons and induces NMDA glutamate receptor hypofunction in Pv-FSI (88). Whether there is some therapeutic benefit in targeting Pv-FSI to mediate addiction-related behaviors have not been fully explored but can be tested by increasing or decreasing Pv-FSI activity in certain brain regions before or after any drug administration.

Studies on substance use disorders have mainly focused on goal-directed and habitual behaviors to explain reward-seeking. Specifically, the striatum has been of particular interest and Pv-FSI have been widely implicated as a target to study reward-seeking because of their powerful ability to inhibit striatal MSNs (72) and involvement in the cortico-striatal circuitry. In fact, activation of Pv-FSI in the mPFC and NAc both result in behavioral changes to reward (37). Research has focuses on Pv-FSI in the cortex (43), and some on the striatum (72) and their role in reward-seeking. One study has found that Pv-FSI in the striatum are involved in reward-conditioned behavioral performance in an experience-dependent manner in which more learning occurs. Moreover, Pv-FSI in the striatum can mediate learning by enhancing performance during associative learning (35). Manipulating Pv-FSI in the striatum to see whether learning of drug reward can be hindered may provide evidence of the importance of Pv-FSI in not just memory but the cue-mediated learning that is needed for drug reinforcement.

*In vivo* and *in vitro* methods using animal models have made it possible to examine the phenomenon of Pv-FSI and connect its molecular and behavior role. *In vivo* methods like optogenetics and chemogenetics approaches such as Designer Receptor Exclusively Activated by Designer Drugs (DREADD) (89) in Cre-recombinase mouse models have been used to investigate the role of Pv-FSI in the mPFC and NAc (43). Such techniques manipulate neuronal firing of Pv-FSI. Using DREADD, one study attempted to elucidate the role of Pv-FSI in alcohol reward-seeking but also how alcohol treatment affected Pv-FSI (43). To explain the molecular mechanism of Pv-FSI and its role in addiction development, they performed *in vivo* microdialysis to assess the changes in GABA and glutamate neurotransmitter levels as a result of Pv-FSI manipulation in the specific brain region (43). Their molecular and behavioral work collectively provides a novel Pv-FSI microcircuit as a fine-tuning mechanism in the corticostriatal circuitry controlling reward-seeking behaviors. Large-scale neural recording and optogenetics results demonstrated that suppressing striatal Pv-FSI reduces the firing of neuronal output and behavior declines with experience (35). These studies demonstrate the use of a combination of powerful technologies to elucidate the multiple roles Pv-FSI can play in a single disease like addiction.

Studies evaluating the downregulation or suppression of Pv-FSI in regard to reward-seeking have been somewhat challenging because of conflicting results that have yet to be disputed. Interestingly, some studies have shown some discrepancy in how Pv-FSI affects compulsive responding and how it may be different depending on the kind of reward. One study showed that striatal FSI ablation did not affect sucrose consumption (72), while another showed activation of Pv-FSI in the NAc decreased compulsive behavior for sucrose rewards (43). Pv-FSI ablation in the dorsal striatum of Pv-Cre transgenic mice attenuates compulsive ethanol consumption (72). Future studies can delve more into why these discrepant functions occur and in different contexts as a way to understand the complexities of addiction and the vulnerabilities of different type of drug abuse substances.

## Future Perspectives

Collectively, Pv-FSI research thus far have demonstrated the amazingly unique properties of Pv-FSI that require 3 crucial components for proper functioning: physiological, chemical/molecular, and cognitive-behavioral. Pv proteins, cell substrates, and constituents are what allow for proper physiological functioning of these cells such as gamma oscillations, fast and high frequency burst firing of inhibitory signals, which have been shown to subsequently affect cognitive functioning. However, the detailed molecular mechanisms of these causal roles are not well-defined. In addition, the brain region-specific functions of these interneurons appear to be different as well. Thus, future studies should be directed to identify the mechanisms of different abnormalities of Pv-FSI and how they are specific to disease in order to test potential therapeutic strategies to target Pv-FSI for disease treatment. The properties and functions of Pv-FSI are multi-faceted, which means there are many opportunities to manipulate different aspects of Pv-FSI activity to resolve gaps in current understanding of these interneurons and in the context of health and disease. Current research has only partly dissected the molecular and behavioral roles of Pv-FSI pertaining to cognitive dysfunction in psychiatric disorders. More research that emphasize the connection between observed molecular and behavioral patterns of Pv-FSI modulation can ascend to discerning the underlying pathological mechanisms of psychiatric disorders and therefore, the development of effective treatment. Further, research can and should be conducted using the well-established models that have proved to be efficient in elucidating the role of Pv-FSI and the mechanisms of their detrimental role in various disorders. Some suggested.

One area of interest that has potential to gain momentum are brain neuroimaging. Such studies conducted on human psychiatric populations utilized proton magnetic resonance spectroscopy (^1^H-MRS), which is optimized for GABA detection, positron emission tomography (PET), and single photon emission computed tomography (SPECT). A systematic review with meta-analysis of these neuroimaging studies of GABA in schizophrenia reflects the urgent need of more studies to develop bigger data sets; this can help address the significant high levels of heterogeneity and thus potentially identify biomarkers of diseases with GABA dysfunction and disease subgroups (90).

While many preclinical studies have utilized only either *in vitro* or *in vivo* techniques, advancements in Pv-FSI research should use a combination of the two. Aside from qPCR, fluorescence-activated cell sorting (FACS), and electrophysiology to identify properties of Pv-FSI, *in vivo* techniques such as optogenetics and chemogenetics, paired with electrophysiology and behavioral approaches can aid in relating the physiological and molecular role of Pv-FSI to the cognitive-behavioral role. A novel *in vivo* approach would be to manipulate molecular/chemical properties of Pv-FSI and assessing the consequential changes in their physiological and cognitive-behavioral functions. This can be done, for example, by manipulating neuronal firing of Pv-FSI in a cell-and brain-specific manner using DREADD and simultaneously obtaining real-time neurochemical or physiological changes in the brain using microdialysis or EEG (43), all while the mice concurrently undergo cognitive-behavioral paradigms such as those for assessing learning and memory. Such combination of techniques can be used to strengthen the understanding of the causal relationship between Pv-FSI molecular activity and functional behaviors based on brain regions. The extent of the feasibility and accuracy of combining these techniques to observe simultaneous changes in brain and behavior is unknown and can be a hurdle to overcome, but worthwhile to optimize.

That is not to say, however, that there will not be further challenges to anticipate despite performing these potential studies with rigor and innovation. Many studies have shown conflicting results addressing the same question regarding Pv-FSI function, but more has occurred when researching in the context of disease. More studies need to look at possible confounding factors such as feedback signals that may dictate the neuronal and behavioral responses. One reason why conflicting evidence may occur is because disease pathology in general is complex, with many different predispositions, and courses of the disease that may change the trajectory of it, including the neuronal and behavioral responses during the course of the disease.

Furthermore, studies regulating Pv-FSI function in disease will be a critical step to develop pharmacological interventions mediated by Pv-FSI. Thus, the combined techniques should also be used to perform rescue experiments in psychiatric disease mouse models to assess the therapeutic potential of targeting Pv-FSI. Since disrupted E/I balance is implicated diseases such as schizophrenia, autism spectrum disorders, Alzheimer's disease, and drug addiction, certainly Pv-FSI can play a a role to mediate the effects and rectify these changes in the brain. GABA-mediated glutamate balance by Pv-FSI may improve disease conditions that are characterized by a disrupted NMDAR activity. For example, increasing Pv-FSI activity may resolve the hyperglutamatergic condition that occurs in alcohol withdrawal such that more neuronal firing of GABA by Pv-FSI may balance the abnormal increases in glutamate. Increasing Pv can also be a strategy to test in schizophrenia mouse models to overcome the reduction of Pv expression which has been associated with schizophrenia symptoms. On the other hand, decreasing Pv can possibly alleviate phenotypes of anxiety and the increased Pv expression exhibited in chronic stress mouse models.

Therefore, to understand Pv-FSI-mediated brain function and dysfunction, it is imperative to continue elucidating the fundamental properties of Pv-FSI, and possible roles of genes that contributes diseases. Regarding physiological properties, Dehorter et al. identified that Er81 transcriptionally controls Kv1.1 expression in Pv-FSI and regulates the intrinsic firing properties of these interneurons. This study uniquely demonstrated the relationship between Pv-FSI's physiological and molecular properties in that conditional mutations in transcriptional regulators not only affect intrinsic features of Pv-FSI postsynaptic neural activity, but also their presynaptic connectivity to receive inputs properly (91). These findings might explain the mechanism of how Pv-FSI fine-tune their activity to adapt to the changing environment. However, whether or not the spike delay and impaired synaptic communication is a direct consequence of Er81-mediated protein expression is still unclear. Moreover, many research have identified other transcription factors that regulate multipotent progenitor cells for the development of specific interneurons, their subtypes, and their specific cell fates (4, 6). Therefore, another opportunity for advancing Pv-FSI research would be to consider the relationship between the proper development of Pv-FSI interneurons and to what extent they contribute to psychiatric diseases. Moreover, the limited understanding of the consequences of improper Pv development in embryonic development and its long-term consequences on the adult brain further warrant the need for researching Pv-FSI related developmental diseases.

The combination of recombinant adeno-associated virus (rAAV) and neuron-specific gene regulation tremendously improves neuroscience research in identifying molecular mechanisms and suggesting treatment targets for psychiatric disorders. Current studies identify short regulatory elements that restrict viral expression to certain brain regions or a select subpopulation of neurons (92). *Scn1a*, a new enhancer that distinctly controls Pv-FSI functioning, may shed some light on potential clinical applications for psychiatric disorders. A combination of AAV with an expression-dependent *Scn1a* enhancer demonstrated that *Scn1a* expression is associated with cortical Pv-FSI function. Moreover, this viral targeting of Pv-FSI using *Scn1a* allows for the selective targeting and manipulation of the cortex, and is effective across mice, non-human primates, and humans. Overall, this study demonstrates that viral manipulation of selected *Scn1a* enhancer may be a possible clinical application targeting Pv-FSI circuits and can be a promising therapeutic mechanism for psychiatric disorders implicating Pv-FSI.

In addition, other avenues that warrant researching Pv-FSI further are those that offer a biochemical perspective. Since Pv-FSI demand high energy for normal functioning, scientists have attributed their neuronal dysfunctions to a mitochondrial deficit. One study that examined the effects of mitochondrial impairment in Pv-FSI by deleting the cox10 gene, important for mitochondrial biosynthesis pathway, selectively in Pv-FSI of mice found that these mice had impaired sociability and sensory gating (93). As oxidative stress can contribute to mitochondrial deficits, one hypothesis is that oxidative stress is the pathological mechanism causing impairments in Pv-FSI exhibited in schizophrenia and other psychiatric disorders (94). However, it is also questionable whether the disorders occur prior to mitochondrial dysfunction or that the dysfunction gives rise to the disorder.

Although, Pv-FSI research to date have provided valuable knowledge and predictions about their roles in the brain and disease, there still remains many puzzling concerns that demonstrate a “chicken or the egg” causality dilemma pertaining to Pv-FSI functions and changes in their local environment. However, examining the causality of these findings (i.e., changes in genetic and synaptic activity) and evaluating the time course and manner of consequential disruption of Pv-FSI in disease would strongly benefit the search for pathophysiological mechanisms for disease.

Overall, research on Pv-FSI thus far has proved that Pv-FSI are a valuable and indispensable potential pharmacological target to research psychiatric conditions. Pv-FSI have many attributes going beyond molecular and behavioral functions that allow for in-depth and interdisciplinary research. Recent research highlight some new and feasible targets that can help unravel mechanisms of Pv-FSI functioning such as ion channels and genes that support their cell firing and neurotransmission. However, future studies need to incorporate these targets and elaborate on findings in the context of disease. Therefore, research that are evolving and promising are those that can relate Pv-FSI constituents to disease by assessing how their physiological, molecular/chemical, and cognitive-behavioral properties are interrelated. These studies will provide a better understanding of not only Pv-FSI but also their etiological and pathogenic involvement underlying brain dysfunctions in disorders, and possible targets for treatment. Future experiments must take advantage of using a combination of tools and techniques to study the role of Pv-FSI with a holistic approach that can give rise to translational research and eventually treat brain disorders.

## Author Contributions

HN contribute for the conceptualization and performed the literature review. LN, BD, and HN wrote the manuscript. All authors read and approved the submitted version.

## Conflict of Interest

The authors declare that the research was conducted in the absence of any commercial or financial relationships that could be construed as a potential conflict of interest.

## Abbreviations

DA: dopamine
GABA: γ-aminobutyric acid
mPFC: medial prefrontal cortex
MSNs: medium spiny neurons
NAc: nucleus accumbens
Pv-FSI: Parvalbumin expressing fast-spiking interneurons
NFSI: Non-fast spiking interneurons
ADI: adapting interneurons
ISI: irregular spiking interneurons
IBI: intrinsic bursting interneurons
ACI: accelerating interneurons
Pv: parvalbumin
SOM: somatostatin
NPY: neuropeptide Y
VIP: vasoactive intestinal peptide
CCK: cholecystokinin.
